# Perception of pragmatic skills by speech therapists and parents of children with autism spectrum disorder

**DOI:** 10.1590/2317-1782/e20240354en

**Published:** 2026-02-09

**Authors:** Marcos Henrique Borges, Valeriana de Castro Guimarães, Fernanda Dreux Miranda Fernandes, Angelina Emiliano Oliveira, Ivone Felix Sousa

**Affiliations:** 1 Curso de Fonoaudiologia, Escola de Ciências Sociais e da Saúde, Pontifícia Universidade Católica de Goiás – PUC-Goiás - Goiânia (GO), Brasil.; 2 Programa de Pós-graduação em Ciências da Saúde, Faculdade de Medicina, Universidade Federal de Goiás – UFG - Goiânia (GO), Brasil.; 3 Faculdade de Medicina, Universidade de São Paulo – USP - São Paulo (SP), Brasil.; 4 Curso de Psicologia, Escola de Ciências Sociais e da Saúde, Pontifícia Universidade Católica de Goiás – PUC-Goiás – Goiânia (GO), Brasil.

**Keywords:** Autism, Speech, Language, Social communication disorder, Autism spectrum disorder

## Abstract

**Purpose:**

To analyze the perceptions of speech therapists and parents or guardians regarding the performance of pragmatic skills of children with autism spectrum disorder (ASD), between 2 and 12 years old, undergoing speech therapy.

**Method:**

Cross-sectional, prospective, observational, analytical study, conducted in a speech therapy clinic in the Midwestern Region of Brazil, including two groups and convenience sampling: Group 1. nine speech therapists who were caring for 70 children with ASD, in the established age range; Group 2. 70 parents or guardians or caregivers of these children. Participants responded to the Protocol for the Assessment of Pragmatic Skills of Children with Autism Spectrum Disorders (PAHPEA). Descriptive analyses were conducted to identify the children's pragmatic difficulties perceived by the two groups, Mann-Whitney U test to compare the perceptions of participants in the two groups about the children's pragmatic skills, and Spearman rho correlational analysis to verify the occurrence of an association between these perceptions of participants in both groups.

**Results:**

A statistically significant difference was found in three of the five factors covered by PAHPEA (responsiveness, functionality, and inadequacy), as the members of Group 1 interpreted the children's performance differently from that of the participants in Group 2.

**Conclusion:**

Overall, the perceptions of speech therapists and parents or guardians were similar in almost all factors and questions evaluated.

## INTRODUCTION

Autism Spectrum Disorder (ASD) is a neurobiological condition that impairs the social, communicative, and behavioral development of the individuals affected by it. It is characterized by deficits in social interaction, verbal and non-verbal communication, and restricted and repetitive patterns of behavior^(1)^.

The prevalence of ASD has been increasing significantly in recent decades^(2,3)^. In 2020, approximately one in 36 children aged 8 years was diagnosed with ASD in 11 regions of the United States^(4)^. In Brazil, it is estimated that about 1% to 2% of the child population is diagnosed with this disorder, which represents a major challenge for the health and education systems^(5)^.

Pragmatic skills constitute a fundamental component of human communication, involving the appropriate use of language in diverse social contexts. Children with well-developed pragmatic skills are able to participate effectively in social interactions, adjusting their language and behavior to social and contextual expectations. This development is mediated by various daily interactions, through which children learn to adequately interpret and respond to social cues^(6)^.

The most current edition of the Diagnostic and Statistical Manual of Mental Disorders DSM-5-TR^TM^, published in March 2022 with its revised text, specifies that social communication (pragmatic) impairments must be present in the diagnosis of ASD.^(7)^ Children with ASD frequently face significant challenges in pragmatic skills, which can result in substantial difficulties in both their communication and social interactions. Furthermore, these pragmatic limitations can lead to social isolation and negatively affect their emotional and cognitive development^(8)^. Pragmatic impairments, which concern the social use of language, have a significant impact on the development of language, speech, and communication in children with ASD, as they often show delays in the development of expressive and receptive language. Even when they have an adequate vocabulary and intact grammatical skills, they may present difficulties in conversational skills, or in understanding social implications and using language in a flexible and adaptive manner^(9)^.

The perception of the impact of social communication disorders and their relationship with the diagnosis of ASD^(10)^ has been discussed for more than two decades. The lack of mastery in this type of communication can culminate in awkward or misinterpreted social interactions. Since social interaction is a communication process, it depends on communicative skills^(11)^. Thus, the lack of repertoire for non-verbal communication can further aggravate difficulties in social interaction and affect the effective communication of children with ASD^(12)^. As a result, these individuals may face even more obstacles in establishing and maintaining social relationships, potentially appearing indifferent or even disinterested, or they may not know how to initiate social interactions or how to respond appropriately when in contact with others^(13)^.

The Protocol for the Assessment of Pragmatic Skills of Children with Autism Spectrum Disorder (PAHPEA) is a tool created in Brazilian Portuguese specifically to evaluate these children^(14)^. This protocol aims to provide a structured and comprehensive approach to assessing the ability of children with ASD to use language appropriately in social contexts, allowing for the identification of specific areas of difficulty so that more precise interventions can be suggested and implemented^(15)^.

There may be significant divergences between the perceptions of speech therapists and parents or guardians or caregivers regarding the pragmatic skills of children with ASD. From a scientific perspective, investigating the perceptions of speech therapists and parents or guardians or caregivers offers a comprehensive understanding of the needs and challenges faced by children with ASD in the development of pragmatic skills^(16)^.

The integrated understanding of the observations of speech therapists and parents or guardians or caregivers regarding the communication skills of children with ASD can provide valuable information to enhance the therapeutic plan and guide the development of intervention, making it more effective and adapted to the individual needs of each child. The collaboration between speech therapists and parents or guardians or caregivers is particularly important, as the latter play a central role in applying therapeutic strategies in the children’s daily lives, providing them with a continuous support environment^(17)^.

The effectiveness of communication between speech therapists and parents or guardians or caregivers is an essential factor for the success of interventions, but there is evidence that it is not always ideal. Studies have justified this assertion by revealing that parents or guardians or caregivers of children with ASD repeatedly feel that their concerns are not completely understood or even valued by the speech therapists who attend to them^(17,18)^.

Therefore, the objective of this study was to analyze, through the application of the PAHPEA, the perceptions of speech therapists and parents or guardians or caregivers regarding the performance of pragmatic skills in children with ASD, aged between 2 and 12 years, who are undergoing speech therapy.

## METHOD

### Study design and location

This was a cross-sectional, prospective, observational, and analytical study, conducted in a private speech therapy clinic, a reference in the care of children with ASD for over 20 years, in Goiânia, GO, in the Midwestern Region of Brazil.

### Ethical Procedures

The project was reviewed and approved by the Research Ethics Committee (CAAE 76548623.7.0000.0037). The researchers scheduled a time and provided an explanation of the study to the speech-language pathologists at a clinic, located in Goiânia, GO, who were treating children with an ASD diagnosis, as well as to their parents or guardians or caregivers. All who chose to participate in this research signed the Free and Informed Consent Form (FICF). This study is in accordance with Brazilian law.^(19)^.

### Population and sample

The study population was constituted by convenience sampling, comprising two groups, regardless of sex:

Group 1. Composed of nine Speech-Language Pathologists, with time since graduation varying between 2 and 9 years, with a mean of 5 years. All had at least one year of experience in providing care to children with ASD. The 70 participating children, as per the protocol guideline, were aged between 2 and 12 years. The tested group had a mean age of 6 years (72 months), had been undergoing speech therapy with the same professional for at least one year, and had a prior diagnosis of ASD. The diagnosis was made by neurologists and/or psychiatrists before the start of the research.

**Group 2.** Comprised 70 parents, guardians, or caregivers of the same 70 children with ASD, who were the primary caregivers. Regarding the level of education, the majority (64.9%) had completed higher education, 17.9% had completed high school, 3.4% had elementary education (early or late years), and 12.8% did not respond. Data collection was performed at a private clinic specializing in Speech-Language Pathology, located in Goiânia (GO), between May and June 2024.

### Inclusion criteria

The following were included: a. Speech-Language Pathologists with at least one year of experience in treating children with a medical diagnosis of ASD. b. Parents or guardians, aged over 21 years, of children with ASD, aged between 2 and 12 years.

### Exclusion criteria

Speech-Language Pathologists, parents or guardians, or caregivers who did not complete their respective questionnaires and/or who withdrew their consent for participation were excluded. Children whose medical diagnosis included comorbidities such as speech apraxia, intellectual disability, Down syndrome, cerebral palsy, among others, were also excluded.

### Data collection instrument

The data collection instrument used was the PAHPEA (Protocol for the Assessment of Pragmatic Skills of Children with Autism Spectrum Disorder), developed for Brazilian Portuguese to cover psychometric parameters regarding the pragmatic performance of children with ASD^(14)^ (Appendix 1). This is a questionnaire composed of 29 questions, answered by members of Group 1 and Group 2 in a location defined by the participants. The protocol involves five factors: intentionality, responsiveness, language, functionality, and inadequacy. All participants responded to the same protocol, indicating their answers on a Likert-type scale, as follows: always = 3, sometimes = 2, and never = 1.

### Data analysis

To meet the objective of the study, descriptive analysis of the data was performed, using frequency distribution for categorical variables and analysis of central tendency and dispersion measures for continuous variables. Descriptive analyses were conducted to identify the main pragmatic difficulties of children with ASD perceived by the members of Group 1 and Group 2. The Mann-Whitney U test was used to compare the perceptions of participants in Group 1 and Group 2 regarding the pragmatic skills of children with ASD and to show whether there was a statistically significant difference between them^(20)^. Spearman’s rho correlational analysis was performed to verify whether there was an association between the perceptions of participants in Group 1 and Group 2 in relation to the responses to the PAHPEA for the assessment of social communication skills in children with ASD^(20)^.

## RESULTS

Based on the data presented in Table 1, a statistically significant difference (p = 0.046) was found for the responsiveness factor, in question 8 [Responds to complex questions (why did he/she do that? What did you do at school?...)], demonstrating that, from the perspective of Group 1 members (mean rank = 76.67) regarding this construct, the children showed better performance than observed by Group 2 participants (mean rank = 64.33).

**Table 1:** Analysis of perceptions of speech therapists (Group 1) and parents or guardians or caregivers (Group 2) regarding the main pragmatic difficulties faced by children diagnosed with autism spectrum disorder (n = 70)

**Pragmatic Skills Factors**	**Group**	**Mean Rank**	**Mann-Whitney U Test**	**Z**	***p***
1. Looks at the adult	Group 1	72.00	2345.00	-0.535	0.593
	Group 2	69.00			
2. Interacts with the adult	Group 1	71.65	2369.50	-0.412	0.681
	Group 2	69.53			
3. Primarily uses speech to communicate	Group 1	75.89	2073.00	-1.701	0.088
	Group 2	65.11			
4. Primarily uses non-verbal sounds to communicate	Group 1	73.32	2252.50	-0.913	0.361
	Group 2	67.68			
5. Primarily uses gestures to communicate	Group 1	73.01	2274.00	-0.850	0.395
	Group 2	67.99			
6. Is easily understood	Group 1	70.79	2430.00	-0.098	0.922
	Group 2	70.21			
7. Responds to simple questions (where is the car? What do you want?...)	Group 1	68.46	2307.50	-0.646	0.519
	Group 2	72.54			
8. Responds to complex questions (why did he/she do that? What did you do at school?...)	Group 1	76.67	2018.00	-1.992	**0.046**
	Group 2	64.33			
9. Responds with isolated words or two-word phrases	Group 1	68.69	2323.00	-0.580	0.562
	Group 2	72.31			
10. Responds with complete sentences with complex structures	Group 1	75.20	2121.00	-1.529	0.126
	Group 2	65.80			
11. Interacts to request actions or objects	Group 1	67.14	2215.00	-1.111	0.267
	Group 2	73.86			
12. Requests information	Group 1	74.39	2177.50	-1.213	0.225
	Group 2	66.61			
13. Makes appropriate comments	Group 1	74.74	2153.00	-1.337	0.181
	Group 2	66.26			
14. Uses isolated words and two-word phrases to communicate	Group 1	72.76	2292.00	-0.725	0.469
	Group 2	68.24			
15. Uses complete sentences and complex structures to communicate	Group 1	72.66	2298.50	-0.696	0.486
	Group 2	68.34			
16. Gives commands	Group 1	68.69	2323.50	-0.564	0.572
	Group 2	72.31			
17. Expresses pleasure fear or displeasure clearly	Group 1	61.20	1799.00	-3.065	**0.002**
	Group 2	79.80			
18. Takes communicative turns appropriately	Group 1	70.53	2448.00	-0.009	0.992
	Group 2	70.47			
19. Engages in pretend play	Group 1	75.36	2109.50	-1.555	0.120
	Group 2	65.64			
20. Makes it clear when he/she does not want to do something appropriately	Group 1	70.50	5450.00	0.000	1.000
	Group 2	70.50			
21. Uses crying. tantrums. or aggression when frustrated or to interrupt an activity	Group 1	80.92	1420.50	-3.448	**0.001**
	Group 2	60.08			
22. Produces decontextualized or non-functional speech, sounds, or gestures	Group 1	79.62	1811.50	-2.911	**0.004**
	Group 2	61.38			
23. Initiates communication	Group 1	72.14	2335.50	-0.514	0.607
	Group 2	68.86			
24. Tells stories or relates facts	Group 1	70.54	2447.00	-0.014	0.989
	Group 2	70.46			
25. Comments on what is happening or may happen (going to fall.... one. two, one more..)	Group 1	69.60	2387.00	-0.283	0.777
	Group 2	71.40			
26. Includes the adult in play	Group 1	65.60	2074.00	-1.820	0.069
	Group 2	75.87			
27. Plays in isolation in repetitive activities	Group 1	77.54	1957.50	-2.287	**0.022**
	Group 2	63.46			
28. Is attentive and understands facial expressions and prosody	Group 1	69.01	2346.00	-0.478	0.633
	Group 2	71.99			
29. Uses facial expressions and prosodic variations to express him/herself	Group 1	69.52	2381.50	-0.329	0.742
	Group 2	71.48			
Interactivity	Group 1	69.37	2371.00	-0.330	0.741
	Group 2	71.63			
Responsiveness	Group 1	73.85	2215.50	-0.997	0.319
	Group 2	67.15			
Language	Group 1	77.16	1983.50	-1.971	**0.049**
	Group 2	63.84			
Functionality	Group 1	70.70	2436.00	-0.059	0.953
	Group 2	70.30			
Inadequacy	Group 1	83.74	1523.50	-3.947	**0.000**
	Group 2	57.26			

Note: The Mean Rank and the Mann-Whitney U test were employed

Regarding the functionality factor, in question 17 (Expresses pleasure, fear, or displeasure clearly), there was a statistically significant difference (p = 0.002), indicating that Group 2 members (mean rank = 79.80) interpreted the children’s performance better compared to Group 1 members (mean rank = 61.20).

For the inadequacy factor, statistically significant differences were observed in responses 21 (Uses crying, tantrums, or aggression when frustrated or to interrupt an activity, with p = 0.001), 22 (Produces decontextualized or non-functional speech, sounds, or gestures, with p = 0.004), and 27 (Plays in isolation, in repetitive activities, with p = 0.022).

In all three cases, Group 1 members interpreted the children’s performance differently from that of Group 2 participants. The conceptions identified by Group 1 and Group 2 members as the main pragmatic difficulties faced by children with ASD can be observed in more detail in Table 1.

Summarizing, according to the analysis results presented in Table 1, Group 2 members more frequently indicated the children’s manifestations of ASD concerning expressing pleasure, fear, or displeasure clearly. Group 1 participants, on the other hand, reported more behaviors of crying, tantrums, or aggression when an activity is interrupted or when the child is frustrated. In addition, Group 1 members indicated more frequently than Group 2 that children with ASD produced more decontextualized or non-functional speech, sounds, or gestures and engaged in isolated play in repetitive activities, as well as that they were capable of answering complex questions. Regarding the other behaviors concerning the pragmatic development of children with ASD evaluated using the PAHPEA, there was no statistically significant difference, demonstrating that both Group 1 and Group 2 participants observed them in the same way.

According to the perceptions of Group 1 and Group 2 members, the main pragmatic difficulties of children with ASD were primarily using speech to communicate, responding to simple questions, and interacting to request actions or objects. When evaluating the factors that comprise pragmatic skills (interactivity, responsiveness, language, functionality, and inadequacy), it was found that Group 1 members better described the development of language and inadequacy skills than Group 2 members.

The results of the Spearman’s rho correlational analysis, used to verify whether there was a relationship between the perceptions of Group 1 and Group 2 participants regarding their responses to the PAHPEA for the assessment of social communication skills in children with ASD, are presented in Table 2. It was detected that the PAHPEA showed a positive correlation between its constituent factors, demonstrating that there was a significant association (p = 0.000) between the perceptions of Group 1 and Group 2 members when observing the pragmatic skills and competences of children with ASD. Furthermore, it was found that the strength of these relationships can be considered moderate (*r* = |0.40 – 0.69|) to strong (*r* = |0.70 – 0.89|). It was also evident that functionality showed the strongest association with interactivity and responsiveness (Table 2).

**Table 2:** Correlation between the perceptions of speech therapists (Group 1) and parents or guardians or caregivers (Group 2) regarding the pragmatic skills factors of children diagnosed with autism spectrum disorder (n = 70)

**Pragmatic Skill Factors**	**Correlation and significance**	**Pragmatic Skill Factors**				
		**Interactivity**	**Responsiveness**	**Language**	**Functionality**	**Inadequacy**
**Interactivity**	r	1.000				
	*p*					
**Responsiveness**	r	0.708^**^	1.000			
	*p*	0.000				
**Language**	r	0.594^**^	0.627^**^	1.000		
	*p*	0.000	0.000			
**Functionality**	r	0.847^**^	0.779^**^	0.637^**^	1.000	
	*p*	0.000	0.000	0.000		
**Inadequacy**	r	0.474^**^	0.429**	0.530^**^	0.453^**^	1.000
	*p*	0.000	0.000	0.000	0.000	

The Spearman rho correlational analysis was used

** *p* ≤ 0.001

**Caption:***r*, correlation; *p*, significance

## DISCUSSION

The data from this study are corroborated by the findings of other works, which also pointed to the possibility of significant divergences between the perceptions of speech therapists and parents or guardians regarding the pragmatic skills of children with ASD^(15)^, or which verified the significant occurrence of communicative deficits in children with ASD from a clinical and family perspective^(21)^.

Additionally, the data from the present study reinforce the observation that parents or guardians are quite capable of perceiving the evolution of communicative skills and, consequently, the improvement in the functionality of their children's communication with ASD in relation to communicative profile, interpersonal and non-interpersonal functions, which can be further enhanced after a speech therapy guidance program^(22)^.

In this study, there was convergence between the interpretation of speech therapists and that of parents or guardians concerning the pragmatic skills of children with ASD. Of the 29 questions analyzed and answered by both groups, only five showed a statistically significant divergence (*p* > 0.5), corresponding to 17% of the total. The discrepancies were observed in the **interactivity** parameter, specifically in the question about the child's eye contact with the adult, and in the **functionality** parameter, involving responses such as requesting information, producing comments, issuing commands, and observations about present or future events. Such differences can be attributed to the professionals' higher demands regarding the quality and intensity of the observed responses.

Of the 29 questions analyzed and answered by both groups, only five showed a statistically significant divergence (p > 0.5), corresponding to 17% of the total. The discrepancies were observed in the **interactivity** parameter, specifically in the question about the child's eye contact with the adult, and in the **functionality** parameter, involving responses such as requesting information, producing comments, issuing commands, and observations about present or future events. Such differences can be attributed to the professionals' higher demands regarding the quality and intensity of the observed responses.

Therefore, integrating the understanding of the perceptions of speech therapists and parents or guardians regarding the communication skills of children with ASD can provide valuable information to enhance existing programs and guide the development of new, more effective intervention methods and programs adapted to the individual needs of these children^(16)^.

It was also evident in the present study that the participation of parents or guardians in the process of evaluating and monitoring the therapeutic evolution of their children with ASD can positively contribute to the process. Some works have highlighted the concern of parents or guardians, mentioning that their concerns are not always fully understood or even considered by speech therapists^(17,18)^. Thus, involving parents or guardians in the speech therapy intervention processes of their children, giving them the possibility to participate in the evaluation and monitoring, can enhance good results.

The personal experiences of parents or guardians, including their level of knowledge about ASD and their own communicative skills, can influence their perceptions of their children's pragmatic development^(23)^.

Reinforcing the need to integrate parents or guardians into the treatment of children with ASD, a scoping review was conducted with the aim of identifying and grouping results obtained globally on the parental perspective of functioning research and summarizing them using the International Classification of Functioning, Disability and Health – Children and Youth Version, seeking the relationship between this classification, the place, and the circumstances in which people live. It was concluded that both the place and the circumstances can have a significant influence on the perception of daily life functioning ^(24)^.

Another point to highlight, but which was not evaluated in the present study, is that cultural factors can also influence both the expectations and perceptions about the development of pragmatic skills in children with ASD. However, most existing studies on the topic focus on populations in developed countries, which leaves a significant gap in the understanding of perceptions in different cultural and socioeconomic contexts^(14,24)^.

The development of communicative skills and competences impacts social communication (pragmatic) performance. Consequently, the ineffectiveness in using these skills can trigger disruptive behaviors. This study indicated that parents or guardians interpreted the responses of their children with ASD as inadequate, in questions 21, 22, and 27, which encompass the inadequacy factor.

Understanding the children’s communication skills can decrease anxiety and increase the confidence of parents or guardians in their ability to support them. When parents or guardians are well-informed and involved in the therapeutic process, they begin to understand the strategies and techniques used by speech therapists, becoming active partners in the intervention process. In this way, interventions are generally more effective, and the progress of children with ASD can be more significant and sustainable^(17,18)^.

## CONCLUSION

There was significant convergence between the perceptions of parents and speech therapists regarding the pragmatic skills of children with ASD, with agreement exceeding 75% in the responses to the PAHPEA. This convergence highlights the importance of the parental perspective as a complementary and valuable source for the assessment of social communication, especially regarding functionality and responsiveness, which are essential factors for pragmatic development. The present findings corroborated previous studies that highlighted the parents’ ability to identify changes in their children’s communication, particularly when included in speech therapy guidance and monitoring programs.

Despite the general agreement, it was found that speech therapists more frequently identified difficulties related to inadequacy, such as the use of decontextualized behaviors, tantrums, and isolated play, while parents reported more behaviors related to emotional expression and communicative functionality. These differences may reflect the parents’ greater exposure to the children’s daily behavior, while the professionals adopt a more technical and structured approach during the assessment.

The strong correlation among the PAHPEA factors reinforces the interdependence among pragmatic skills, indicating that the development of communicative functionality is closely associated with interactivity and responsiveness. This datum highlights the need for interventions that include the integration of multiple pragmatic skills, promoting not only communicative competence but also social engagement and self-regulation in children with ASD.

The relevance of active parental participation in the assessment and intervention process is emphasized, showing that their inclusion can enhance therapeutic outcomes. Parental involvement, combined with an interaction-centered approach and respect for individual perceptions, contributes to a more holistic understanding of the child's needs and to the construction of more personalized and effective intervention strategies.

Considering the potential influence of cultural and socioeconomic factors on perceptions about pragmatic skills, it is recommended that future studies extend the investigation to different contexts, aiming to deepen the understanding of the diversity of family experiences and needs. This approach can contribute to the development of intervention programs that are more inclusive and sensitive to the particularities of each community.

The PAHPEA can improve the identification and treatment of pragmatic deficits in children with ASD, enabling the development and application of more effective and collaborative intervention strategies for pragmatic skills.

In summary, this study reinforces the importance of integrating the perspectives of parents and professionals in the assessment of pragmatic skills in children with ASD, proposing a collaborative assessment and intervention model that values both technical knowledge and the daily experience of caregivers. This integrated approach can represent a significant advance in promoting social communication and improving the quality of life for children with ASD and their families.

## Appendix 1 Protocol for the Assessment of Pragmatic Skills of Children with Autism Spectrum Disorder (PAHPEA)

This protocol aims to characterize various pragmatic skills of children with ASD. The score should primarily be used to monitor the clinical evolution of each child and not as a criterion for comparison between children.

Child’s Name: ________________________________________________________ Name of the Speech Therapist, or Parent/Mother, or Guardian, or Caregiver: ___________ Child’s Date of Birth: ___________ Child’s Age: _____ (between 2 and 12 years)

QUESTIONNAIRE TO BE ANSWERED BY SPEECH THERAPISTS, PARENTS OR GUARDIANS OR CAREGIVERS

Answer based on your experience with the child over the last six months.

**Table:**

	**Always**	**Sometimes**	**Never**
	**3**	**2**	**1**
1. Looks at the adult			
2. Interacts with the adult			
3. Primarily uses speech to communicate			
4. Primarily uses non-verbal sounds to communicate			
5. Primarily uses gestures to communicate			
6. Is easily understood			
7. Responds to simple questions (where is the car? what do you want?...)			
8. Responds to complex questions (why did he/she do that? what did you do at school?...)			
9. Responds with isolated words or two-word phrases			
10. Responds with complete sentences with complex structure			
11. Interacts to request actions or objects			
12. Requests information			
13. Makes appropriate comments			
14. Uses isolated words and two-word phrases to communicate			
15. Uses complete sentences and complex structures to communicate			
16. Give commands			
17. Expresses pleasure, fear, or displeasure clearly			
18. Takes communicative turns appropriately			
19. Engages in pretend play			
20. Makes it clear when he/she does not want to do something appropriately			
21. Uses crying, tantrums, or aggression when frustrated or to interrupt an activity			
22. Produces decontextualized or non-functional speech, sounds, or gestures			
23. Initiates communication			
24. Tells stories or relates fact			
25. Comments on what is happening or may happen (going to fall..., one, two, one more...			
26. Includes the adult in play			
27. Plays in isolation, in repetitive activities			
28. Is attentive and understands facial expressions and prosody			
29. Uses facial expressions and prosodic variations to express him/herself			

**Table:**

Interactivity	Language	**Functionality**	Responsiveness	Inadequacy
9 questions	5 questions	8 questions	4 questions	3 questions

**Source:** Based on Fernandes^(14)^
