# Supplementary material for: Perception of pragmatic skills by speech therapists and parents of children with autism spectrum disorder
Source: Codas. 2026 Feb 9;38(1):e20240354. doi: 10.1590/2317-1782/e20240354en (PMC12978319; doi:10.1590/2317-1782/e20240354en)
Supplement: Translation

# Translation: Percepção das habilidades pragmáticas por fonoaudiólogos e pais de crianças com transtorno do espectro do autismo

**DOI:** 10.1590/2317-1782/e20240354pt

**Authors:** Marcos Henrique Borges, Valeriana de Castro Guimarães, Fernanda Dreux Miranda Fernandes, Angelina Emiliano Oliveira, Ivone Felix Sousa

**Affiliations:** 1 Curso de Fonoaudiologia, Escola de Ciências Sociais e da Saúde, Pontifícia Universidade Católica de Goiás – PUC-Goiás - Goiânia (GO), Brasil.; 2 Programa de Pós-graduação em Ciências da Saúde, Faculdade de Medicina, Universidade Federal de Goiás – UFG - Goiânia (GO), Brasil.; 3 Faculdade de Medicina, Universidade de São Paulo – USP - São Paulo (SP), Brasil.; 4 Curso de Psicologia, Escola de Ciências Sociais e da Saúde, Pontifícia Universidade Católica de Goiás – PUC-Goiás – Goiânia (GO), Brasil.

**Keywords:** Autismo, Fala, Linguagem, Transtorno da Comunicação Social, Transtorno do Espectro do Autismo

## Abstract

**Objetivo:**

Analisar as percepções de fonoaudiólogos/as e pais ou responsáveis acerca do desempenho das habilidades pragmáticas de crianças com transtorno do espectro do autismo (TEA), entre 2 e 12 anos, em acompanhamento fonoaudiológico.

**Método:**

Estudo transversal, prospectivo, observacional, analítico, conduzido em clínica particular de fonoaudiologia na Região Centro-Oeste do Brasil, incluindo dois grupos, por conveniência: Grupo 1. nove fonoaudiólogos/as que estavam atendendo 70 crianças com TEA, na faixa etária estabelecida; Grupo 2. 70 pais ou responsáveis ou cuidadores/as destas crianças. Os participantes responderam ao Protocolo de Avaliação de Habilidades Pragmáticas de Crianças com Transtorno do Espectro do Autismo (PAHPEA). Foram conduzidas análises descritivas para identificar as dificuldades pragmáticas das crianças percebidas pelos dois grupos, teste U de Mann-Whitney para comparar as percepções dos participantes dos dois grupos sobre as habilidades pragmáticas das crianças e análise correlacional Spearman rho para verificar se houve associação entre estas percepções dos participantes dos dois grupos.

**Resultados:**

Houve diferença estatisticamente significativa de forma pontual em três dos cinco fatores abarcados pelo PAHPEA (responsividade, funcionalidade e inadequação), pois os integrantes do Grupo 1 interpretaram o desempenho das crianças de forma diferente daquela dos participantes do Grupo 2.

**Conclusão:**

No geral, as percepções de fonoaudiólogos/as e pais ou responsáveis se aproximaram em quase todos os fatores e questões avaliados, houve convergência significativa com concordância superior à 75% nas respostas ao PAHPEA.

## INTRODUÇÃO

O transtorno do espectro do autismo (TEA) é uma condição neurobiológica que prejudica o desenvolvimento social, comunicativo e comportamental das pessoas por ele afetadas. É caracterizado por déficits em interação social, comunicação verbal e não verbal e por padrões de comportamento restritivos e repetitivos^(1)^.

A prevalência do TEA vem aumentando significativamente nas últimas décadas^(2,3)^. Em 2020, aproximadamente uma em cada 36 crianças de 8 anos de idade foi diagnosticada com TEA em 11 regiões dos Estados Unidos^(4)^. No Brasil, estima-se que cerca de 1% a 2% da população infantil seja diagnosticada com esse transtorno, o que representa grande desafio para os sistemas de saúde e educação^(5)^.

As habilidades pragmáticas constituem componente fundamental da comunicação humana, envolvendo a utilização adequada da linguagem em contextos sociais diversos. Crianças com habilidades pragmáticas bem desenvolvidas são capazes de participar efetivamente de interações sociais, ajustando sua linguagem e comportamento às expectativas sociais e contextuais. Esse desenvolvimento é mediado por diversas interações cotidianas, por meio das quais as crianças aprendem a interpretar e a responder adequadamente aos sinais sociais^(6)^.

A edição mais atual do *Diagnostic and statistical manual of mental disorders DSM-5-TR^TM^* , publicada em março de 2022 com seu texto revisado, especifica que as alterações da comunicação social (pragmática) devem estar presentes no diagnóstico do TEA^(7)^. Frequentemente, crianças com TEA enfrentam desafios significativos nas habilidades pragmáticas, o que pode resultar em dificuldades substanciais tanto em sua comunicação quanto em suas interações sociais^(8)^. Adicionalmente, essas limitações pragmáticas podem levar ao isolamento social e afetar negativamente seu desenvolvimento emocional e cognitivo^(9)^.

As alterações pragmáticas, que dizem respeito ao uso social da linguagem, têm impacto significativo no desenvolvimento de linguagem, fala e comunicação em crianças com TEA^(10)^, pois amiúde demonstram atrasos no desenvolvimento da linguagem expressiva e receptiva. Mesmo quando possuem vocabulário adequado e habilidades gramaticais intactas, podem apresentar dificuldades em habilidades conversacionais, ou para compreender implicações sociais e utilizar a linguagem de maneira flexível e adaptativa^(11)^.

Há mais de duas décadas, a percepção do impacto dos transtornos da comunicação social e a sua relação com o diagnóstico de TEA têm sido discutidas. A falta de domínio nesse tipo de comunicação pode culminar em interações sociais desajeitadas ou mal interpretadas. Como a interação social é um processo de comunicação, depende das habilidades comunicativas. Desse modo, a falta de repertório para a comunicação não verbal pode agravar ainda mais as dificuldades em convívio social e afetar a comunicação eficaz das crianças com TEA^(12)^. Como resultado, esses indivíduos podem enfrentar ainda mais obstáculos para estabelecer e manter relacionamentos sociais, podendo parecer indiferentes ou até mesmo desinteressados, ou podem não saber como dar início a interações sociais ou como responder adequadamente quando em contato com outras pessoas^(13)^.

O Protocolo de Avaliação de Habilidades Pragmáticas de Crianças com Transtorno do Espectro do Autismo (PAHPEA) é uma ferramenta criada em português do Brasil especificamente para avaliar essas crianças^(14)^. Esse protocolo visa fornecer abordagem estruturada e abrangente para avaliar a capacidade das crianças com TEA de usar a linguagem em contextos sociais de forma adequada, permitindo identificar áreas específicas de dificuldade de modo que possam ser sugeridas e implementadas intervenções mais precisas^(15)^.

Pode haver divergências significativas entre as percepções de fonoaudiólogos/as e pais ou responsáveis ou cuidadores/as sobre as habilidades pragmáticas de crianças com TEA. Do ponto de vista científico, a investigação das percepções de fonoaudiólogos/as e pais ou responsáveis ou cuidadores/as oferece compreensão abrangente sobre as necessidades e os desafios enfrentados por crianças com TEA no desenvolvimento de habilidades pragmáticas^(16)^.

O entendimento integrado acerca das observações de fonoaudiólogos/as e pais ou responsáveis ou cuidadores/as sobre as habilidades de comunicação de crianças com TEA pode fornecer informações valiosas para aprimorar o plano terapêutico e direcionar o desenvolvimento da intervenção, tornando-a mais eficaz e adaptada às necessidades individuais de cada criança. A colaboração entre fonoaudiólogos/as e pais ou responsáveis ou cuidadores/as é particularmente importante, pois estes últimos desempenham papel central na aplicação das estratégias terapêuticas no cotidiano das crianças, proporcionando-lhes ambiente de apoio contínuo^(17)^.

A efetividade da comunicação entre fonoaudiólogos/as e pais ou responsáveis ou cuidadores/as é fator essencial para o sucesso das intervenções, mas há evidências de que ela nem sempre é ideal. Estudos justificaram essa afirmação ao revelar que pais ou responsáveis ou cuidadores/as de crianças com TEA reiteradamente sentem que suas preocupações não são completamente entendidas ou mesmo valorizadas pelos/as fonoaudiólogos/as que as atendem^(17,18)^.

Diante disso, este estudo teve por objetivo analisar, por meio da aplicação do PAHPEA, as percepções de fonoaudiólogos/as e de pais ou responsáveis ou cuidadores/as acerca do desempenho em relação às habilidades pragmáticas de crianças com TEA, com idades entre 2 e 12 anos, que estão em acompanhamento fonoaudiológico.

## MÉTODO

### Tipo de estudo e local

Trata-se de um estudo transversal, prospectivo, observacional, analítico, realizado em uma clínica particular de fonoaudiologia, referência no atendimento de crianças com TEA há mais de 20 anos, em Goiânia, GO, na Região Centro-Oeste do Brasil.

### Procedimentos éticos

O projeto desta pesquisa foi apreciado e aprovado pelo Comitê de Ética em Pesquisa (CAAE 76548623.7.0000.0037). Os pesquisadores agendaram horário e fizeram uma explanação sobre o presente estudo para os profissionais de fonoaudiologia de uma clínica, localizada em Goiânia, GO, que estavam atendendo crianças com diagnóstico de TEA, assim como para seus pais ou responsáveis ou cuidadores/as. Todos que optaram por participar desta pesquisa assinaram o Termo de Consentimento Livre e Esclarecido (TCLE). Este estudo está em conformidade com a legislação brasileira^(19)^.

### População e amostra

A população do estudo foi constituída por conveniência, composta de dois grupos, independentemente de sexo: Grupo 1. Composto por nove profissionais de Fonoaudiologia, com tempo de formação variando entre 2 e 9 anos, sendo a média de 5 anos. Todos possuíam, no mínimo, um ano de experiência no atendimento a crianças com TEA. As 70 crianças participantes do estudo, conforme orientação do protocolo, possuíam idades entre 2 e 12 anos. O grupo testado tinha idade média de 6 anos (72 meses), estes estavam em acompanhamento fonoaudiológico com o mesmo profissional há pelo menos um ano e possuíam diagnóstico prévio de TEA. O diagnóstico foi realizado por médicos neurologistas e/ou psiquiatras antes do início da pesquisa. Grupo 2. Participaram 70 pais, responsáveis ou cuidadores das mesmas 70 crianças com TEA, sendo estes os principais cuidadores. Em relação ao nível de escolaridade, a maioria (64,9%) possuía ensino superior completo, 17,9% tinham ensino médio completo, 3,4% tinham ensino fundamental (anos iniciais ou finais), e 12,8% não responderam. A coleta de dados foi realizada em uma clínica particular especializada em Fonoaudiologia, localizada em Goiânia (GO), entre maio e junho de 2024.

### Critérios de inclusão

Foram incluídos: a. fonoaudiólogos/as com experiência em atendimento a crianças com diagnóstico médico de TEA há pelo menos um ano; b. pais ou responsáveis, com idade superior a 21 anos, de crianças com TEA, com idades entre 2 e 12 anos.

### Critérios de exclusão

Foram excluídos fonoaudiólogos/as, pais ou responsáveis ou cuidadores/as que não completaram seus respectivos questionários e/ou que retiraram seu consentimento para participação. Crianças que apresentavam no diagnóstico médico comorbidades tais como apraxia de fala, deficiência intelectual, síndromes de Down, paralisia cerebral, entre outros.

### Instrumento de coleta

O instrumento de coleta de dados utilizado foi o PAHPEA, desenvolvido para português do Brasil com a finalidade de abarcar parâmetros psicométricos sobre o desempenho pragmático de crianças com TEA^(14)^ (Apêndice 1). Trata-se de questionário composto de 29 perguntas, respondido pelos componentes do Grupo 1 e do Grupo 2 em local definido pelos participantes. O protocolo envolve cinco fatores: intencionalidade, responsividade, linguagem, funcionalidade e inadequação. Todos os participantes responderam ao mesmo protocolo, indicando suas respostas em uma escala de tipo Likert, da seguinte maneira: sempre = 3, às vezes = 2 e nunca = 1.

### Análise dos dados

Para atender ao objetivo do estudo, foi realizada a análise descritiva dos dados, por meio da distribuição de frequência das variáveis categóricas e análise das medidas de tendência central e de dispersão das variáveis contínuas.

A fim de identificar as principais dificuldades pragmáticas de crianças com TEA percebidas pelos componentes do Grupo 1 e do Grupo 2, foram conduzidas análises descritivas. Para comparar as percepções dos participantes do Grupo 1 e do Grupo 2 sobre as habilidades pragmáticas de crianças com TEA e evidenciar se houve diferença estatística significativa entre elas, foi empregado o teste U de Mann-Whitney^(20)^. Com o intuito de verificar se houve associação entre as percepções dos participantes do Grupo 1 e do Grupo 2 em relação às respostas ao PAHPEA para a avaliação das habilidades de comunicação social de crianças com TEA, foi realizada a análise correlacional Spearman rho^(20)^.

## RESULTADOS

Pelos dados apresentados na Tabela 1, verifica-se que houve diferença estatística significativa (*p* = 0,046) quanto ao fator responsividade, na questão 8 [Responde a perguntas complexas (porque ele/a fez isso? o que você fez na escola?...)], demonstrando que, do ponto de vista dos componentes do Grupo 1 (média de rank = 76,67) acerca deste construto, as crianças apresentaram melhor desempenho que na observação dos participantes do Grupo 2 (média de rank = 64,33).

**Tabela 1:** Análise das percepções de fonoaudiólogos/as (Grupo 1) e pais ou responsáveis ou cuidadores/as (Grupo 2) acerca das principais dificuldades pragmáticas enfrentadas por crianças com diagnóstico de transtorno do espectro do autismo (n = 70)

**Habilidade pragmática**	**Grupo**	**Média de rank**	**Teste U de Mann-Whitney**	**Z**	***p***
1. Olha para o adulto	Grupo 1	72,00	2345,00	-0,535	0,593
	Grupo 2	69,00			
2. Interage com o adulto	Grupo 1	71,65	2369,50	-0,412	0,681
	Grupo 2	69,53			
3. Usa principalmente a fala para se comunicar	Grupo 1	75,89	2073,00	-1,701	0,088
	Grupo 2	65,11			
4. Usa principalmente sons não verbais para se comunicar	Grupo 1	73,32	2252,50	-0,913	0,361
	Grupo 2	67,68			
5. Usa principalmente gestos para se comunicar	Grupo 1	73,01	2274,00	-0,850	0,395
	Grupo 2	67,99			
6. Se faz entender facilmente	Grupo 1	70,79	2430,00	-0,098	0,922
	Grupo 2	70,21			
7. Responde a perguntas simples (cadê o carrinho? O que você quer?...)	Grupo 1	68,46	2307,50	-0,646	0,519
	Grupo 2	72,54			
8. Responde a perguntas complexas (porque ele/a fez isso? o que você fez na escola?...)	Grupo 1	76,67	2018,00	-1,992	**0,046**
	Grupo 2	64,33			
9. Responde com palavras isoladas ou frases com duas palavras	Grupo 1	68,69	2323,00	-0,580	0,562
	Grupo 2	72,31			
10. Responde com frases completas com estruturas complexas	Grupo 1	75,20	2121,00	-1,529	0,126
	Grupo 2	65,80			
11. Interage para pedir ações ou objetos	Grupo 1	67,14	2215,00	-1,111	0,267
	Grupo 2	73,86			
12. Pede informações	Grupo 1	74,39	2177,50	-1,213	0,225
	Grupo 2	66,61			
13. Faz comentários adequados	Grupo 1	74,74	2153,00	-1,337	0,181
	Grupo 2	66,26			
14. Usa palavras isoladas e frases de duas palavras para se comunicar	Grupo 1	72,76	2292,00	-0,725	0,469
	Grupo 2	68,24			
15. Usa frases completas e estruturas complexas para se comunicar	Grupo 1	72,66	2298,50	-0,696	0,486
	Grupo 2	68,34			
16. Dá ordens	Grupo 1	68,69	2323,50	-0,564	0,572
	Grupo 2	72,31			
17. Expressa prazer, medo ou descontentamento de forma clara	Grupo 1	61,20	1799,00	-3,065	**0,002**
	Grupo 2	79,80			
18. Troca turnos comunicativos de forma adequada	Grupo 1	70,53	2448,00	-0,009	0,992
	Grupo 2	70,47			
19. Brinca de faz de conta	Grupo 1	75,36	2109,50	-1,555	0,120
	Grupo 2	65,64			
20. Deixa claro quando não quer fazer alguma coisa de forma adequada	Grupo 1	70,50	5450,00	0,000	1,000
	Grupo 2	70,50			
21. Usa choro, birra ou agressão quando frustrada ou para interromper alguma atividade	Grupo 1	80,92	1420,50	-3,448	**0,001**
	Grupo 2	60,08			
22. Produz fala, sons ou gestos descontextualizados ou não funcionais	Grupo 1	79,62	1811,50	-2,911	**0,004**
	Grupo 2	61,38			
23. Inicia comunicação	Grupo 1	72,14	2335,50	-0,514	0,607
	Grupo 2	68,86			
24. Conta histórias ou relata fatos	Grupo 1	70,54	2447,00	-0,014	0,989
	Grupo 2	70,46			
25. Comenta sobre o que está acontecendo ou pode acontecer (vai cair..., um, dois, mais um..)	Grupo 1	69,60	2387,00	-0,283	0,777
	Grupo 2	71,40			
26. Inclui o adulto na brincadeira	Grupo 1	65,60	2074,00	-1,820	0,069
	Grupo 2	75,87			
27. Brinca isolada, em atividades repetitivas	Grupo 1	77,54	1957,50	-2,287	**0,022**
	Grupo 2	63,46			
28. É atenta e compreende expressões faciais e prosódia	Grupo 1	69,01	2346,00	-0,478	0,633
	Grupo 2	71,99			
29. Usa expressões faciais e variações prosódicas para se expressar	Grupo 1	69,52	2381,50	-0,329	0,742
	Grupo 2	71,48			
Interatividade	Grupo 1	69,37	2371,00	-0,330	0,741
	Grupo 2	71,63			
Responsividade	Grupo 1	73,85	2215,50	-0,997	0,319
	Grupo 2	67,15			
Linguagem	Grupo 1	77,16	1983,50	-1,971	**0,049**
	Grupo 2	63,84			
Funcionalidade	Grupo 1	70,70	2436,00	-0,059	0,953
	Grupo 2	70,30			
Inadequação	Grupo 1	83,74	1523,50	-3,947	**0,000**
	Grupo 2	57,26			

A Média de rank e o teste U de Mann-Whitney foram empregados

Em relação ao fator funcionalidade, na questão 17 (Expressa prazer, medo ou descontentamento de forma clara), houve diferença estatística significativa (*p* = 0,002), indicando que os componentes do Grupo 2 (média de rank = 79,80) interpretaram melhor o desempenho das crianças em comparação com os membros do Grupo 1 (média de rank = 61,20).

Para o fator inadequação, foram observadas diferenças estatisticamente significativas nas respostas 21 (Usa choro, birra ou agressão quando frustrada ou para interromper alguma atividade, com *p* = 0,001), 22 (Produz fala, sons ou gestos descontextualizados ou não funcionais, com *p* = 0,004) e 27 (Brinca isolada, em atividades repetitivas, com *p* = 0,022).

Nos três casos, os integrantes do Grupo 1 interpretaram o desempenho das crianças de forma diferente daquela dos participantes do Grupo 2. As concepções identificadas pelos componentes do Grupo 1 e do Grupo 2 como sendo as principais dificuldades pragmáticas enfrentadas pelas crianças com TEA podem ser observadas com mais detalhes na Tabela 1.

Sintetizando, de acordo com os resultados das análises apresentados na Tabela 1, observa-se que os membros do Grupo 2 indicaram com maior frequência as manifestações das crianças com TEA com relação a expressar prazer, medo ou descontentamento de forma clara. Já os participantes do Grupo 1 relataram mais comportamentos de choro, birra ou agressão quando se interrompe uma atividade ou quando a criança é frustrada. Em adição a isso, os membros do Grupo 1 indicaram mais que os do Grupo 2 que as crianças com TEA produziram mais falas, sons ou gestos descontextualizados ou não funcionais e brincadeiras isoladas em atividades repetitivas, assim como que elas eram capazes de responder perguntas complexas. Já em relação aos demais comportamentos concernentes ao desenvolvimento pragmático das crianças com TEA avaliados por meio do PAHPEA, não houve diferença estatisticamente significativa, demonstrando que tanto os participantes do Grupo 1 quanto os do Grupo 2 os observaram da mesma forma.

Conforme as percepções dos componentes do Grupo 1 e do Grupo 2, as principais dificuldades pragmáticas das crianças com TEA foram usar principalmente a fala para se comunicar, responder a perguntas simples e interagir para pedir ações ou objetos. Ao avaliar os fatores que compõem as habilidades pragmáticas (interatividade, responsividade, linguagem, funcionalidade e inadequação), verificou-se que os integrantes do Grupo 1 descreveram melhor o desenvolvimento das habilidades de linguagem e inadequação que os membros do Grupo 2.

Os resultados da análise correlacional Spearman rho, empregada para verificar se houve relação entre as percepções dos participantes do Grupo 1 e do Grupo 2 quanto às suas respostas ao PAHPEA para a avaliação das habilidades de comunicação social de crianças com TEA, encontram-se na Tabela 2. Detectou-se que o PAHPEA apresentou correlação positiva entre os fatores que o compõem, demonstrando que houve associação significativa (*p* = 0,000) entre as percepções dos componentes do Grupo 1 e do Grupo 2 ao observar as habilidades e as competências pragmáticas das crianças com TEA. Além disso, constatou-se que a força dessas relações pode ser considerada de moderada (r = |0,40 – 0,69|) a forte (r = |0,70 – 0,89|)^(21)^. Também evidenciou-se que a funcionalidade apresentou associação mais forte com a interatividade e a responsividade (Tabela 2).

**Tabela 2:** Correlação entre as percepções de fonoaudiólogos/as (Grupo 1) e pais ou responsáveis ou cuidadores/as (Grupo 2) acerca dos fatores das habilidades pragmáticas de crianças com diagnóstico de transtorno do espectro do autismo (n = 70)

**Fatores das habilidades pragmáticas**	**Correlação e significância**	**Fatores das habilidades pragmáticas**				
		**Interatividade**	**Responsividade**	**Linguagem**	**Funcionalidade**	**Inadequação**
**Interatividade**	r	1,000				
	*p*					
**Responsividade**	r	0,708^**^	1,000			
	*p*	0,000				
**Linguagem**	r	0,594^**^	0,627^**^	1,000		
	*p*	0,000	0,000			
**Funcionalidade**	r	0,847^**^	0,779^**^	0,637^**^	1,000	
	*p*	0,000	0,000	0,000		
**Inadequação**	r	0,474^**^	0,429**	0,530^**^	0,453^**^	1,000
	*p*	0,000	0,000	0,000	0,000	

A análise correlacional Spearman rho foi utilizada

** *p* ≤ 0,001 **Legenda:** r, correlação; *p*, significância

## DISCUSSÃO

Os dados deste estudo são corroborados pelos achados de outros trabalhos, que também apontaram a possibilidade de haver divergências significativas entre as percepções de fonoaudiólogos/as e pais ou responsáveis acerca das habilidades pragmáticas de crianças com TEA^(15)^, ou que verificaram a ocorrência significativa de déficits comunicativos em crianças com TEA do ponto de vista clínico e familiar^(21)^.

Adicionalmente, os dados do presente estudo reforçam a observação de que pais ou responsáveis são bastante capazes de perceber a evolução das habilidades comunicativas e, consequentemente, a melhora da funcionalidade da comunicação de suas crianças com TEA em relação a perfil comunicativo, funções interpessoais e não interpessoais, o que pode ser ainda mais aprimorado após um programa de orientação fonoaudiológica^(22)^.

Neste estudo houve convergência entre a interpretação de fonoaudiólogos/as e a de pais ou responsáveis no que concerne às habilidades pragmáticas de crianças com TEA. Das 29 questões analisadas e respondidas por ambos os grupos, apenas cinco apresentaram divergência estatisticamente significativa (p > 0,5), correspondendo a 17% do total. As discrepâncias foram observadas no parâmetro **interatividade**, especificamente na questão sobre o contato visual da criança com o adulto, e no parâmetro **funcionalidade**, envolvendo respostas como solicitação de informações, produção de comentários, emissão de ordens e observações sobre eventos presentes ou futuros. Tais diferenças podem ser atribuídas à maior exigência dos profissionais em relação à qualidade e à intensidade das respostas observadas.

Portanto, a integração do entendimento sobre as percepções de fonoaudiólogos/as e pais ou responsáveis sobre as habilidades de comunicação de crianças com TEA pode fornecer informações valiosas para aprimorar os programas já existentes e direcionar o desenvolvimento de novos métodos e programas de intervenção mais eficazes e adaptados às necessidades individuais destas crianças^(16)^.

Também ficou evidenciado no presente estudo que a participação de pais ou responsáveis no processo de avaliação e acompanhamento da evolução terapêutica de suas crianças com TEA pode colaborar positivamente no processo. Alguns trabalhos deram destaque à inquietação de pais ou responsáveis ao mencionar que suas preocupações nem sempre são totalmente compreendidas ou até mesmo consideradas pelos/as fonoaudiólogos/as^(17,18)^. Desse modo, envolver os pais ou responsáveis nos processos de intervenção fonoaudiológica de suas crianças, dando a eles a possibilidade de participar da avaliação e do acompanhamento, pode potencializar os bons resultados.

As experiências pessoais dos pais ou responsáveis, incluindo seu nível de conhecimento sobre TEA e suas próprias habilidades comunicativas, podem influenciar suas percepções sobre o desenvolvimento pragmático de seus filhos^(23)^.

Reforçando a necessidade de integrar os pais ou responsáveis no tratamento de crianças com TEA, uma revisão de escopo foi conduzida com o intuito de identificar e agrupar resultados obtidos em nível global sobre a perspectiva parental da pesquisa sobre funcionalidade e resumi-los usando a Classificação Internacional de Funcionalidade, Incapacidade e Saúde – Versão Infantil e Juvenil, buscando a relação entre esta classificação, o lugar e as circunstâncias em que as pessoas vivem. Concluiu-se que tanto o lugar quanto as circunstâncias podem ter influência significativa na percepção do funcionamento da vida diária^(24)^.

Outro ponto a destacar, mas que não foi avaliado no presente estudo, é que fatores culturais também podem influenciar tanto as expectativas quanto as percepções sobre o desenvolvimento das habilidades pragmáticas de crianças com TEA. Contudo, a maioria dos estudos existentes sobre o tema concentra-se em populações de países desenvolvidos, o que deixa lacuna significativa na compreensão das percepções em diferentes contextos culturais e socioeconômicos^(14,24)^.

O desenvolvimento das habilidades e competências comunicativas impacta o desempenho da comunicação social (pragmática). Por consequência, a ineficácia no uso dessas habilidades pode desencadear comportamentos disruptivos. Este estudo indicou que os pais ou responsáveis interpretaram as respostas de suas crianças com TEA como inadequadas, nas questões 21, 22 e 27, que abarcam o fator inadequação.

O entendimento sobre as habilidades de comunicação das crianças pode diminuir a ansiedade e aumentar a confiança de pais ou responsáveis em sua capacidade de apoiá-las. Quando pais ou responsáveis são bem informados e envolvidos no processo terapêutico, passam a compreender as estratégias e as técnicas utilizadas pelos/as fonoaudiólogos/as, tornando-se parceiros ativos no processo de intervenção. Desse modo, as intervenções são geralmente mais eficazes e o progresso das crianças com TEA pode ser mais significativo e sustentável^(17,18)^.

## CONCLUSÃO

Houve convergência significativa entre as percepções de pais e fonoaudiólogos acerca das habilidades pragmáticas de crianças com TEA, com concordância superior a 75% nas respostas ao PAHPEA. Essa convergência aponta para a importância da perspectiva parental como fonte complementar e valiosa para a avaliação da comunicação social, especialmente em relação à funcionalidade e responsividade, fatores essenciais para o desenvolvimento pragmático. Os presentes achados corroboraram estudos prévios, que destacaram a capacidade dos pais em identificar mudanças na comunicação de seus filhos, particularmente quando inseridos em programas de orientação e acompanhamento fonoaudiológico.

Apesar da concordância geral, verificou-se que os fonoaudiólogos identificaram mais frequentemente dificuldades relacionadas à inadequação, como o uso de comportamentos descontextualizados, birras e brincadeiras isoladas, enquanto os pais relataram mais comportamentos relacionados à expressão emocional e funcionalidade comunicativa. Essas diferenças podem refletir a maior exposição dos pais ao comportamento cotidiano das crianças, ao passo que os profissionais adotam abordagem mais técnica e estruturada durante a avaliação.

A forte correlação entre os fatores do PAHPEA reforça a interdependência entre as habilidades pragmáticas, indicando que o desenvolvimento da funcionalidade comunicativa está estreitamente associado à interatividade e à responsividade. Esse dado destaca a necessidade de intervenções que contemplem a integração de múltiplas habilidades pragmáticas, promovendo não apenas a competência comunicativa, mas também o engajamento social e a autorregulação das crianças com TEA.

Ressalta-se a relevância da participação ativa dos pais no processo de avaliação e intervenção, evidenciando que sua inclusão pode potencializar os resultados terapêuticos. O envolvimento parental, aliado à abordagem centrada na interação e ao respeito às percepções individuais, contribui para a compreensão mais holística das necessidades da criança e para a construção de estratégias de intervenção mais personalizadas e eficazes.

Considerando a influência potencial de fatores culturais e socioeconômicos nas percepções sobre as habilidades pragmáticas, recomenda-se que estudos futuros ampliem a investigação para diferentes contextos, visando aprofundar a compreensão sobre a diversidade de experiências e necessidades das famílias. Essa abordagem pode contribuir para o desenvolvimento de programas de intervenção mais inclusivos e sensíveis às particularidades de cada comunidade.

O PAHPEA pode melhorar a identificação e o tratamento dos déficits pragmáticos de crianças com TEA, possibilitando o desenvolvimento e a aplicação de estratégias de intervenção nas habilidades pragmáticas mais eficazes e colaborativas.

Em síntese, este estudo reforça a importância de integrar as perspectivas de pais e profissionais na avaliação das habilidades pragmáticas de crianças com TEA, propondo um modelo de avaliação e intervenção colaborativa, que valorize tanto o conhecimento técnico quanto a vivência cotidiana dos cuidadores. Essa abordagem integrada pode representar avanço significativo na promoção da comunicação social e na melhoria da qualidade de vida das crianças com TEA e suas famílias.

## Apêndice 1 Protocolo de Avaliação de Habilidades Pragmáticas de Crianças com Transtorno do Espectro do Autismo (PAHPEA)

Este protocolo visa caracterizar diversas habilidades pragmáticas das crianças com TEA.

A pontuação deve ser usada principalmente para acompanhar a evolução clínica de cada criança e não como critério de comparação entre crianças.

Nome da criança: ________________________________________________________

Nome do/a fonoaudiólogo/a, ou pai/mãe, ou responsável, ou cuidador/a: ___________

Data de nascimento da criança: ___________ Idade da criança: _____ (entre 2 e 12 anos)

QUESTIONÁRIO A SER RESPONDIDO POR FONOAUDIÓLOGOS/AS, PAIS OU RESPONSÁVEIS OU CUIDADORES/AS

Responder com base em sua experiência com a criança no último semestre.

**Table:**

	**Sempre**	**Às vezes**	**Nunca**
	**3**	**2**	**1**
1. Olha para o adulto			
2. Interage com o adulto			
3. Usa principalmente a fala para se comunicar			
4. Usa principalmente sons não verbais para se comunicar			
5. Usa principalmente gestos para se comunicar			
6. Se faz entender facilmente			
7. Responde a perguntas simples (cadê o carrinho? o que você quer?...)			
8. Responde a perguntas complexas (porque ele/a fez isso? o que você fez na escola?...)			
9. Responde com palavras isoladas ou frases de duas palavras			
10. Responde com frases completas com estruturas complexas			
11. Interage para pedir ações ou objetos			
12. Pede informações			
13. Faz comentários adequados			
14. Usa palavras isoladas e frases de duas palavras para se comunicar			
15. Usa frases completas e estruturas complexas para se comunicar			
16. Dá ordens			
17. Expressa prazer, medo ou descontentamento de forma clara			
18. Troca turnos comunicativos de forma adequada			
19. Brinca de faz de conta			
20. Deixa claro quando não quer fazer alguma coisa de forma adequada			
21. Usa choro, birra ou agressão quando frustrada ou para interromper alguma atividade			
22. Produz fala, sons ou gestos descontextualizados ou não funcionais			
23. Inicia comunicação			
24. Conta histórias ou relata fatos			
25. Comenta sobre o que está acontecendo ou pode acontecer (vai cair..., um, dois, mais um...)			
26. Inclui o adulto na brincadeira			
27. Brinca isolada, em atividades repetitivas			
28. É atenta e compreende expressões faciais e prosódia			
29. Usa expressões faciais e variações prosódicas para se expressar			

**Table:**

**Interatividade**	**Linguagem**	**Funcionalidade**	**Responsividade**	**Inadequação**
9 perguntas	5 perguntas	8 perguntas	4 perguntas	3 perguntas

**Fonte:** Baseado em Fernandes^(14)^
